# A systematic review of studies that measure parental vaccine attitudes and beliefs in childhood vaccination

**DOI:** 10.1186/s12889-020-09327-8

**Published:** 2020-08-17

**Authors:** Amalie Dyda, Catherine King, Aditi Dey, Julie Leask, Adam G. Dunn

**Affiliations:** 1 Centre for Health Informatics, Australian Institute of Health Innovation, Macquarie University, Sydney, NSW Australia; 2 Department of Health Systems and Populations, Faculty of Medicine, Health and Human Sciences, Macquarie University, Sydney, NSW Australia; 3 National Centre for Immunisation Research & Surveillance, Sydney, NSW Australia; 4 The University of Sydney, Children’s Hospital at Westmead Clinical School, Faculty of Medicine and Health, Sydney, NSW Australia; 5 The University of Sydney, School of Medicine, Faculty of Medicine and Health, Sydney, NSW Australia; 6 The University of Sydney, Susan Wakil School of Nursing and Midwifery, Sydney, NSW Australia; 7 The University of Sydney, Discipline of Biomedical Informatics and Digital Health, School of Medical Sciences, Faculty of Medicine and Health, Sydney, NSW Australia

**Keywords:** Hesitancy, Refusal, Immunization/immunisation, Vaccination, Vaccines, questionnaire

## Abstract

**Background:**

Acceptance of vaccines is an important predictor of vaccine uptake. This has public health implications as those who are not vaccinated are at a higher risk of infection from vaccine preventable diseases. We aimed to examine how parental attitudes and beliefs towards childhood vaccination were measured in questionnaires through a systematic review of the literature*.*

**Methods:**

We systematically reviewed the literature to identify primary research studies using tools to measure vaccine attitudes and beliefs, published between January 2012 and May 2018. Studies were included if they involved a quantitative survey of the attitudes and beliefs of parents about vaccinations recommended for children. We undertook a synthesis of the results with a focus on evaluating the tools used to measure hesitancy.

**Results:**

A total of 116 studies met the inclusion criteria, 99 used a cross sectional study design, 5 used a case control study design, 4 used a pre-post study design and 8 used mixed methods study designs. Sample sizes of included studies ranged from 49 to 12,259. The most commonly used tool was the Parent Attitudes about Childhood Vaccines (PACV) Survey (*n* = 7). The most common theoretical framework used was the Health Belief Model (*n* = 25). Questions eliciting vaccination attitudes and beliefs varied widely.

**Conclusions:**

There was heterogeneity in the types of questionnaires used in studies investigating attitudes and beliefs about vaccination in parents. Methods to measure parental attitudes and beliefs about vaccination could be improved with validated and standardised yet flexible instruments. The use of a standard set of questions should be encouraged in this area of study.

## Background

Childhood vaccination rates vary widely by country and region, and the reasons for these variations are likely to be context-specific [1–3]. While access to vaccination is a perennial challenge, acceptance also remains an issue of importance to uptake which is affected by an individual’s feelings, attitudes and beliefs about vaccination [4]. There is a spectrum of attitudes towards vaccination, including those who are pro-vaccination and accept all vaccines, those who have many concerns but may fully or partially vaccinate, and those who refuse all vaccines [5]. Those who have questions and concerns have been shown to have lower levels of vaccination uptake [6] which may have a substantial impact on vaccination coverage and increases the risk of outbreaks [7]. Not only are unvaccinated individuals at higher risk of infection and adverse health outcomes, but under-vaccinated populations are at higher risk of more severe outbreaks [8–10].

A range of questionnaires have been developed and tested for measuring vaccination attitudes and beliefs [11]. The largest recent questionnaires in the area include The Vaccine Confidence Project [12] which collected 65,819 responses across 67 countries [13], and the Wellcome Global Monitor 2018 [14], which collected more than 140,000 responses from 140 countries. Both were based on the same set of questions, which included items about vaccine importance, effectiveness, safety, and religious compatibility.

Studies using questionnaires to understand vaccine attitudes and beliefs often modify existing items to incorporate the local context of a specific country or region. There is high variability with respect to use of behavioural theories to inform constructs and items and the comprehensiveness of validation, such as whether the items predict vaccination uptake. Moreover, high variability in how constructs such as vaccine confidence are measured between different questionnaires makes it difficult to assess how attitudes and beliefs vary globally.

Our aim was to examine how parental attitudes and beliefs towards childhood vaccination were measured in questionnaires through a systematic review of the literature.

## Methods

### Inclusion criteria

Studies were included if they were quantitative primary studies investigating parental vaccine attitudes and/or beliefs, regardless of whether they considered one or a combination of vaccines or vaccine-preventable diseases. For the purpose of this review studies on vaccine hesitancy were included, with vaccine hesitancy defined as “a motivational state of being conflicted about, or opposed to, getting vaccinated” [15]. Vaccine hesitancy can result in “a delay in acceptance or refusal of vaccines despite availability of vaccination services” [16]. Studies published after January 2012 were included. Studies were excluded if they investigated vaccination barriers not associated with attitudes or beliefs (e.g. measuring access other than as a factor affecting convenience), adult and adolescent vaccination, or if they were not reported in English. We applied no geographical constraints.

### Search strategy

This review was developed in line with the PRISMA guidelines [17]. Key bibliographic databases were searched to identify relevant articles. The 19 databases searched included: OVID Medline, PsycINFO and Database of Systematic Reviews (see Additional File 1 for the full list of databases searched) Search terms included thesaurus terms (where available) such as ‘Immunization’, ‘Immunization programs’, ‘Vaccines’, ‘Decision Making’, ‘Decision Theory’, ‘Attitude to Health’, ‘Health Behavior’, ‘Risk Assessment’, ‘Trust’, ‘Uncertainty’, ‘Vaccination Refusal’, ‘Anti-Vaccination movement’, ‘Child, Preschool’ and ‘Infant’ These were used with relevant associated text terms. Truncation was utilised to ensure all variant spelling endings of text words were retrieved. The searches were limited to items published from 2012 and ‘Humans’. (see Additional File 1 for the full search strategy). The last search was conducted on 19 May 2018. Articles reviewed for inclusion were limited from January 2012 to May 2018 to avoid duplicating the findings of a 2014 systematic review that reviewed the global literature on vaccine hesitancy [5].

All titles and abstracts or executive summaries found through the search strategy were screened independently by two authors (Adam Dunn and Amalie Dyda) to determine if they were relevant to the review. The full text of those articles that appeared to meet the inclusion criteria were retrieved and reviewed for relevance independently by the same two authors. The reference lists of all included items were searched to identify any additional items for inclusion.

### Data extraction and synthesis

Data were extracted by one author (Amalie Dyda) and confirmed by a second author (Adam Dunn). A standard data extraction form developed by the authors was used. For each study, study design information extracted from the articles included the method of recruitment and the location and type of participants, the number of participants recruited (and completing the study, where appropriate), the vaccine or set of vaccines of relevance to the study, and details of the questions used to measure attitudes and belief about vaccination including any description of behavioural theories used to inform the questionnaire design, and whether the questions were taken directly or adapted from existing instruments. We defined validated questionnaires as those that followed “the process of establishing that a survey item or measure serves the intended purpose. This process can include establishing whether it measures the intended construct using qualitative means (advice from experts, cognitive testing with lay people) and quantitative means (convergent, discriminant, predictive validity)” [18]. Data extracted from each study were tabulated and grouped by study type and study characteristics including sample size, recruitment method, and location.

## Results

**Fig. 1:**
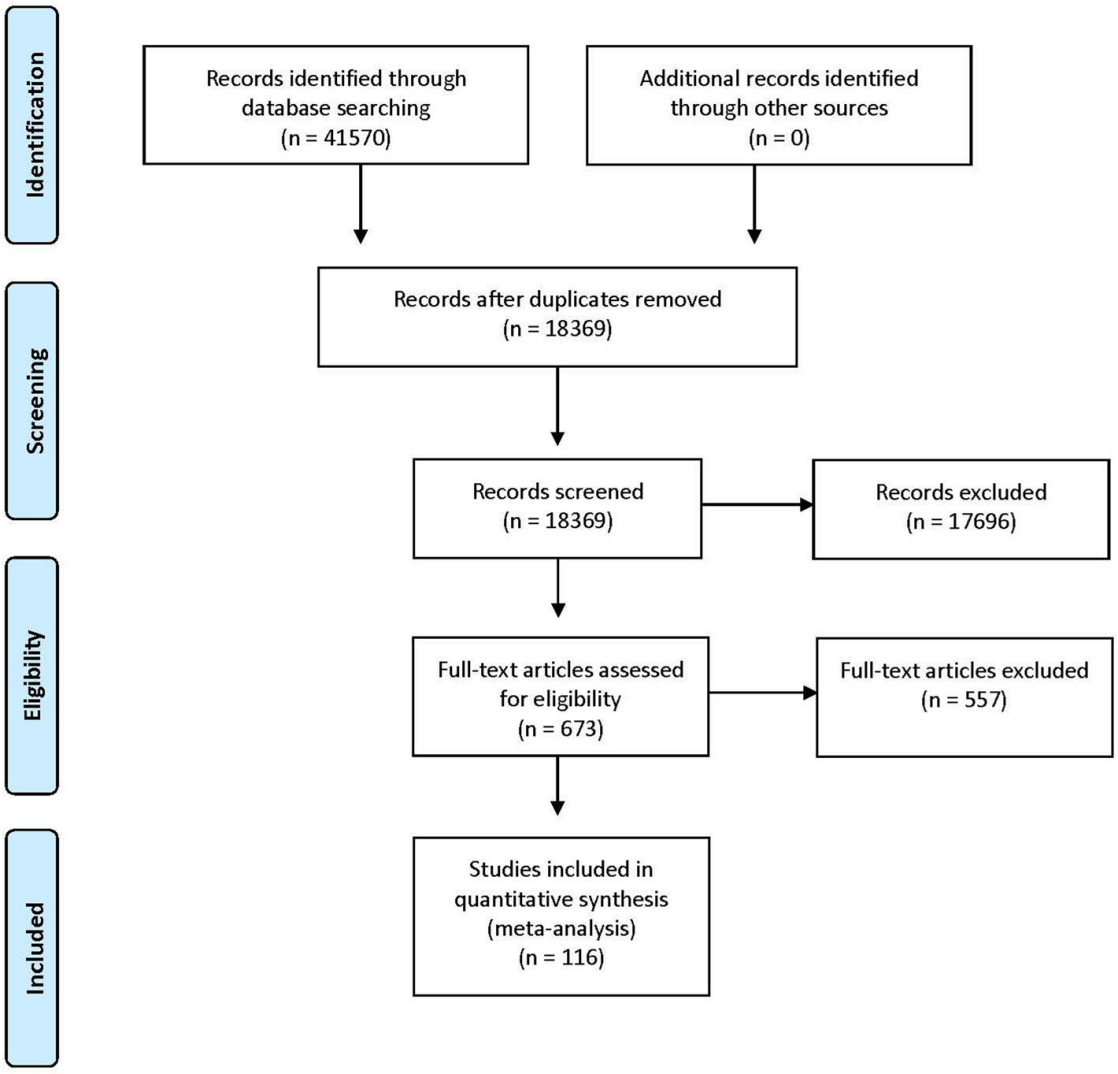
Summary of the search strategy results and set of included studies

The initial search strategy returned 41,570 titles and abstracts, of which 23,201 were removed as duplicates. Title and abstract screening identified 673 full text items for review. Of these, 116 met the inclusion criteria (Fig. 1). A review of the reference lists of included articles did not identify any additional items for inclusion.

### Summary of included studies

Of the included studies, 99 (85.3%) used a cross sectional study design (Additional File 2). Sample sizes across all 116 included studies ranged from 49 to 12,259 participants, with a median of 455 participants. Parental attitudes and beliefs about childhood vaccines in general were studied in 57 (49.1%) studies, and attitudes and beliefs about influenza vaccination (including pandemic H1N1 influenza) in 35 (30.2%). The other 24 (20.7%) studies asked participants about attitudes and beliefs for other specific vaccines, such as polio and rotavirus vaccines.

**Fig. 2:**
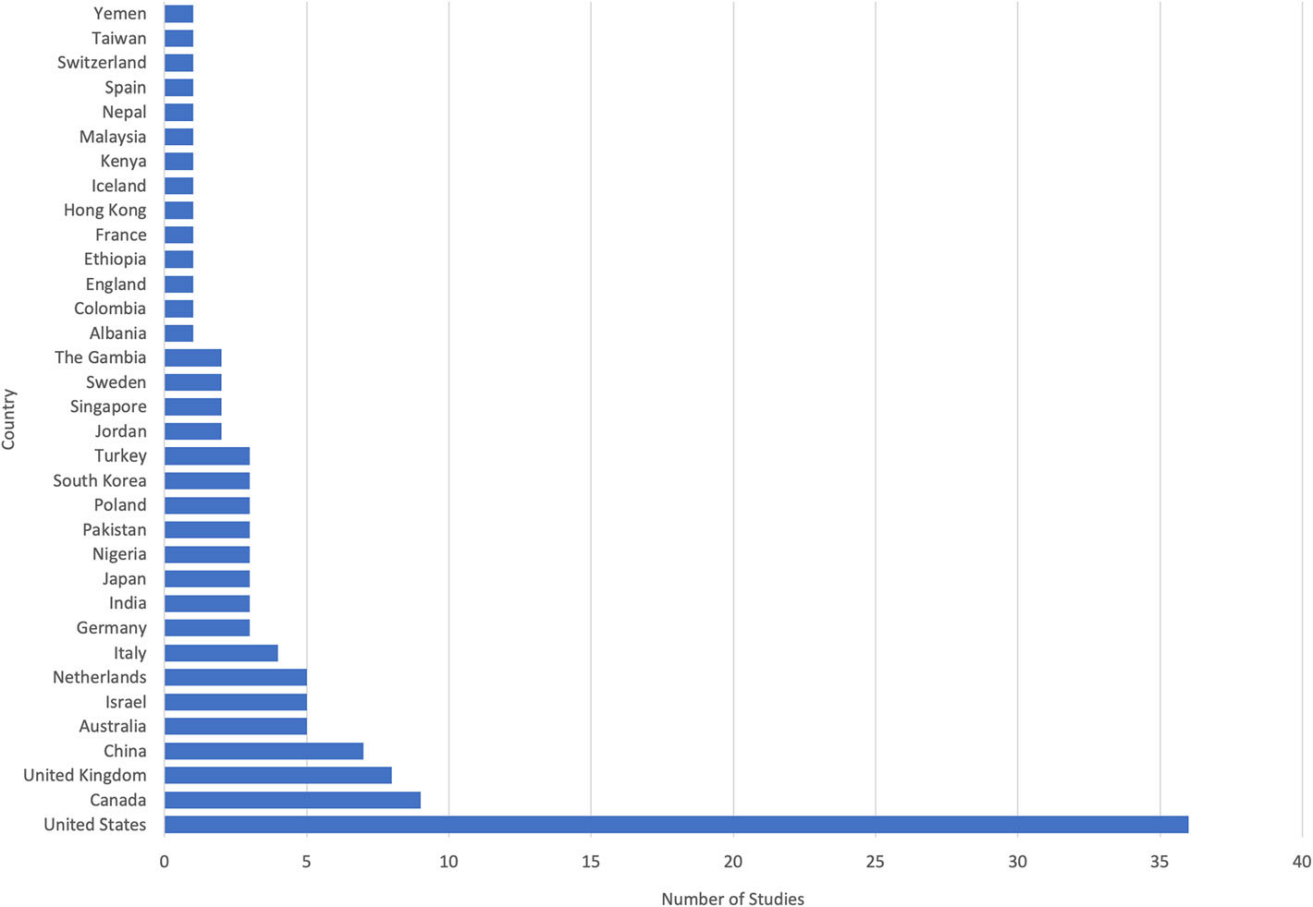
Among the set of 116 included studies, 34 countries were represented

Thirty-four countries were represented in the included studies (Fig. 2). The most common country in which studies were conducted was the United States (*n* = 36), followed by Canada (*n* = 9) and the United Kingdom (*n* = 8). When aggregated by the number of participants, the United States included the largest number (40,155 participants), followed by Canada (7200 participants), and the United Kingdom (3273 participants).

### Questionnaires and survey instruments

One hundred and fourteen studies used a survey design, with the two remaining studies using interviews. The questions asked of participants varied substantially across the set of included studies. There was heterogeneity both in terms of the specific questions asked of participants as well as the provenance of those questions in theory or from standardised questionnaire sets. Sixty three studies reported at least one aspect of validation.

The most commonly used standard questionnaire was the Parent Attitudes about Childhood Vaccines (PACV) Survey Tool (*n* = 7), used in 4 studies with its full format with 15 questions [19–22]. In some studies, the PACV questions were adapted to match the local context or study population, such as in Malaysia [21] and for expectant parents in the United States [19]. In 3 studies, a subset of the PACV questions were used [23–25]. Other questionnaires used included 6 studies based on national immunisation surveys or health department questionnaires [26–31], 1 study based on the Parental Attitudes toward MMR Vaccine and Trust in Medical Authority questionnaire [32], and 1 that used the Vaccine Safety, Attitudes, Training and Communication measures [33].

A total of 62 (53.4%) included studies developed questionnaires using previous literature or previously developed questionnaires, 7 developed questionnaires with experts in the field, 1 used a self-developed scale, and 6 conducted a qualitative data to elicit appropriate questions. The remaining 40 studies did not report having used previous examples as the basis for the designs of their questionnaires.

A variety of theoretical frameworks were used to inform the design of the questionnaires used in the studies. The most common was the Health Belief Model (HBM), which was explicitly stated as having been used to inform the questions in 25 (19.0%) studies [30, 32, 34–57], followed by the Theory of Planned Behaviour, which was used in 5 (4.3%) studies [58–63]. Other studies that were adapted from existing questionnaires may have implicitly been based on these or other theoretical frameworks as a consequence of having adapted from other questionnaires but did not explicitly claim the theoretical framework as a basis for their questions.

### Questions about intention to vaccinate

Of the 116 included studies, 38 (32.8%) included questions in which parents were directly asked about their vaccination intentions for one or more antigens. The specific questions that were asked varied across the set of studies. Examples included, “If you had another infant today, would you want him or her to get all the recommended shots?, “I would get a flu vaccine for my child under 5, every year, if it was free?”, and “If your child were offered it at some point in the future, would you vaccinate them against swine flu?”. This variation precluded a synthesis of the results, and the proportion of participants responding in the affirmative varied substantially across the set of studies.

Of the 38 studies which asked about vaccination intentions for one or more antigens, 16 (13.8%) of these specifically asked about whether they would have children vaccinated for all childhood vaccines. The percentages in these studies ranged from 75% in a study involving 200 parents in the United States [64] to 98% in a study involving 54 parents in Canada [35]. For the 9 (7.8%) studies that asked about intentions in relation to influenza vaccination, the percentages ranged from 29% in a study involving 236 parents in Canada [65] to 92% in a before and after study at a clinic involving 5284 and 5755 different groups of parents in rural Kenya [66].

## Discussion

A substantial number of studies quantitatively examine the childhood vaccination attitudes and beliefs of parents across a broad range of countries. A large number of studies did not report using a validated questionnaire. The countries in which the highest number of studies were conducted were the United States, Canada and the United Kingdom, with most other countries having either none or only a small number of studies. There were significant differences in the way in which questionnaires were developed and the questions asked in each of the studies, making synthesis or comparison of findings a challenge. The use of standardised questionnaires globally would allow findings across countries to be compared and help track longitudinal trends.

The geographical distribution of primary studies included in the review was generally consistent with a previous review on attitudes and beliefs regarding vaccination [5], in which most included studies were conducted in North America and Europe. Among the subset of studies that used standardised questionnaires, there was no clear difference in rates of vaccine hesitancy between countries, nor any clear pattern in the attitudes and beliefs that exhibited the strongest associations with intention. Given that only a relatively small subset used standardised questionnaires, this result is a reflection of the small number of studies rather than evidence of consistency in what matters most to parents exhibiting vaccine hesitancy.

There was little consistency in the provenance of the questions used to measure attitudes and beliefs across studies. A number of studies did not report how the questionnaire or survey instrument was developed, making comparison of these studies difficult. The majority of studies reported construct and item development methods such as basing the questionnaire on previous literature, expert opinion or the use of previously developed surveys.

The use of qualitative evidence is best practice for forming constructs [67] and the use of a previously validated questionnaire is the most appropriate methodology as this ensures that items have content, construct and predictive validity. Previously developed questionnaires which are not validated may not accurately capture information, which is then repeated if these questionnaires are reused [18]. However, as there is no agreed upon gold standard survey instrument, a wide range of sources were used for development, resulting in heterogeneity of questionnaires. The most commonly used standard questionnaire was the PACV Survey Tool, which has been validated in two different settings and been shown to identify vaccine hesitant parents. The questionnaire focuses on the domains of ‘Safety and efficacy’, ‘General attitudes’ and ‘Behaviour’ [68, 69]. The use of this questionnaire for studies investigating vaccine hesitancy should be encouraged to better allow for comparison across studies.

For theoretical frameworks, we found that the HBM was most commonly used to support the development of questionnaires, which was consistent with previous reviews [5]. The HBM posits that perceptions of susceptibility, severity, benefit and barriers, cues to action and self-efficacy predict behaviour. This and other models place emphasis on risk appraisals as important predictors of vaccination. Use of the HBM is complicated by the fact that all related perceptions could apply to vaccination uptake as much as disease outcomes. Since these models look at individual psychological factors by design, they are weaker at measuring other factors like false contraindications, social influence, or access to services or vaccines, which are more likely to be effective in increasing uptake, if they are addressed [15]. Further, many models fail to measure trust, yet trust in vaccination arises as a relevant phenomenon in both qualitative accounts of under-vaccination and the influence of vaccine safety scares [15]. Trust is often “ill-defined and a loosely measured concept” [70]. Recent work on the moral foundations of behaviour suggests that measuring constructs such as contamination and liberty are also relevant [71, 72]. Further work is needed to incorporate moral foundations, other feelings and attitudes and beliefs and trust into a single model of vaccination behaviour and test its robustness.

Future studies in this area may benefit from considering standardised questions on vaccine attitudes and beliefs and other barriers or facilitators [11]. Large international surveys based on a standardised set of questions may be useful for providing international comparisons with context-specific additional questions. To consider the local context, qualitative investigations could supplement the broad based quantitative knowledge from surveys. Both forms of data collection are useful but are also resource intensive and relatively slow to report.

Current outbreaks of measles in the US highlight the importance of monitoring and measuring attitudes and beliefs about vaccinations. From 1st January to 18th July 2019 there were a total of 1148 cases of measles identified in the US which is the largest number of infections reported since 1992. Outbreaks are occurring across a number of states, with an outbreak in Rockland County, reporting the majority (78.4%) of cases have not been vaccinated [73].

The development of the internet has increased the speed with which information and misinformation can spread in the community. This may outpace our ability to measure and report on attitudes and beliefs using current survey methods which are time and resource intensive. Due to the time lag involved, using these methods may limit the ability to support the rapid design of evidence-informed and localised interventions for debunking or mitigating the impact of misinformation.

There were several limitations to the review approach and conduct. The first limitation was that the geographical distribution of the studies included in the review may be biased by the exclusion of studies not written in English. In addition, parental beliefs and attitudes towards influenza vaccination often differ from routine childhood vaccinations [74]. This childhood vaccine was included as some countries recommend annual influenza vaccination, but this is unlikely to affect the findings regarding tools used to monitor attitudes and beliefs about vaccination.

## Conclusion

Despite the number of studies investigating parental attitudes and beliefs about childhood vaccination which were conducted in at least 36 countries, there was heterogeneity in survey designs. Methods to measure parental attitudes and beliefs about vaccination could be improved with validated and standardised yet flexible instruments, supplemented with qualitative investigations. The use of a standard set of validated questions should be encouraged in this area of study to identify, track, and monitor longitudinal trends using quality data.

## Supplementary information

**Additional file 1.** Search strategy. Detailed description of search strategy used for review.

**Additional file 2: Table 1.** Summary of included studies. Summary table of each included study with details about study characteristics.

## Data Availability

Not applicable.

## Abbreviations

HBM: Health belief model
PACV: Parent attitudes about childhood vaccines
