# Supplementary material for: A systematic review of studies that measure parental vaccine attitudes and beliefs in childhood vaccination
Source: BMC Public Health. 2020 Aug 17;20:1253. doi: 10.1186/s12889-020-09327-8 (PMC7433363; doi:10.1186/s12889-020-09327-8)
Supplement: Supplementary file 1 — Additional file 1. Search strategy. Detailed description of search strategy used for review. [file 12889_2020_9327_MOESM1_ESM.docx]

**Additional File 2**

Search Strategy

Databases and dates:

OVID Medline (1946 to March Week 4 2018), OVID Embase (1974 to April 19 2018), PsycINFO (1987 –April Week 2 2018), Cochrane Library databases including Database of Systematic Reviews (Issue 5 of 12, May 2018), Central Register of Controlled Trials (Issue 4 of 12, April 2018), Database of Abstracts of Reviews of Effects (Issue 2 of 4, April 2015), NHS Economic Evaluation Database (Issue 2 of 4, April 2015) and Health Technology Assessments (Issue 4 of 4, October 2016), SCOPUS ( 1823-May 2018) and Web of Science Core Collection including Science Citation Index Expanded (1900-May 2018), Social Sciences Citation Index (1900-May 2018), Arts & Humanities Citation Index (1975-May 2018), Emerging Sources Citation Index (2015-May 2018), Conference Proceedings Citation Index - Science (1990-May 2018), Conference Proceedings Citation Index - Social Sciences & Humanities (1990-May 2018), Book Citation Index– Science (2005-May 2018), Book Citation Index– Social Sciences & Humanities January (2005-May 2018), Current Chemical Reactions (1985-May 2018) and Index Chemicus (1993-May 2018).

OVID Medline

Search Strategy:

--------------------------------------------------------------------------------

1 exp Immunization/

2 exp Immunization Programs/

3 exp Vaccines/

4 (immunis$ or immuniz$ or vaccin$).tw.

5 1 or 2 or 3 or 4

6 exp Decision Making/

7 exp Decision Theory/

8 exp Attitude to Health/

9 exp Health Behavior/

10 exp Risk Assessment/

11 exp Trust/

12 exp Uncertainty/

13 (choice$ or choos$ or decid$ or decis$ or consent$).tw.

14 (attitude$ or knowledge$ or belie$ or view$ or opinion$ or thought$ or think$ or perceive$ or percepti$ or perspective$ or understand$ or prefer$ or trust$).tw.

15 (barrier$ or reason$).tw.

16 (action$ or behav$).tw.

17 (risk$ and (assess$ or perce$ or avers$ or concern$)).tw.

18 (uncertain$ or undecided or hesita$ or doubt$).tw.

19 (refus$ or reject$ or omission$ or omit$ or object or objection).tw.

20 (incomplet$ or delay$ or suboptimal$).tw.

21 6 or 7 or 8 or 9 or 10 or 11 or 12 or 13 or 14 or 15 or 16 or 17 or 18 or 19 or 20

22 5 and 21

23 exp Vaccination Refusal/

24 exp Anti-Vaccination Movement/

25 23 or 24

26 22 or 25

27 limit 26 to humans

28 limit 27 to ("all infant (birth to 23 months)" or "preschool child (2 to 5 years)")

29 exp Child, Preschool/

30 exp Infant/

31 exp Infant, Newborn/

32 (paediatric$ or pediatric$ or child$ or pre-school$ or preschool$ or infant$ or toddler$ or baby or babies).tw.

33 29 or 30 or 31 or 32

34 27 and 33

35 28 or 34

36 limit 35 to yr=2012-2018
