# Supplementary material for: A systematic review of studies that measure parental vaccine attitudes and beliefs in childhood vaccination
Source: BMC Public Health. 2020 Aug 17;20:1253. doi: 10.1186/s12889-020-09327-8 (PMC7433363; doi:10.1186/s12889-020-09327-8)
Supplement: Supplementary file 2 — Additional file 2: Table 1. Summary of included studies. Summary table of each included study with details about study characteristics. [file 12889_2020_9327_MOESM2_ESM.docx]

**Additional File 1**

Table 1: Summary of included studies

| **Article ID** | **Study design** | **Sample size** | **Child age** | **Time period** | **Country** | **Vaccine type** | **Questionnaire developed using** | **Localised** |
| --- | --- | --- | --- | --- | --- | --- | --- | --- |
| Henrikson 2017 (20) | Follow up survey | 237 | 24 months | 2015 | United States | All childhood vaccines | PACV | n/s |
| Orr 2017 (22) | Cross sectional | 86 | 6 months-7 years | Oct Nov 2015-2016 influenza season | United States | Influenza | PACV | n/s |
| Oladejo 2016 (23) | Cross sectional | 1200 | <12 years | N/S | United States | All childhood vaccines | Based on PACV and the HealthStyles survey | n/s |
| Cataldi 2016 (25) | Cross sectional | 351 | <1yr | Apr-Jun 2015 | United States | Measles, Mumps, Rubella | PACV, information from CDC and piloted | n/s |
| Schoeppe 2017 (24) | Pre-post survey | 460 & 238 | N/S | N/S | United States | All childhood vaccines | Some items from PACV and experts | n/s |
| Mohd Azizi 2017 (21) | Cross sectional | 604 | <7 years | Nov-Dec 2016 | Malaysia | All childhood vaccines | Malay-PACV | Translated into Malay |
| Cunningham 2018 (19) | Cross sectional | 648 | N/S | Jul 2014-Sep 2015 | United States | All childhood vaccines | Modified PACV survey | Modified for use among expectant parents |
| Dube 2018 (59) | Cross sectional | 213 | 24-59 months | Mar-15 | Canada | All childhood vaccines | TPB | English and French |
| Dubé 2012 (58) | Longitudinal study | 413 | 0-6 weeks | 2008 – 2009 | Canada | Rotavirus | TPB | English and French |
| Fadel 2017 (60) | Prospective survey | 3268 | 2-6 months | 2011-2014 | United States | All childhood vaccines | TPB | English and Spanish |
| Harmsen 2012 (61) | Cross sectional | 906 | Newborn | N/S | Netherlands | Hepatitis B | TPB | n/s |
| MacDougall 2016 (62) | Before and After study | 722 & 709 | N/S | N/S | Canada | Rotavirus | TPB |  |
| Armitage 2018 (34) | Cross sectional | 454 | N/S | Aug-Sep 2017 | The Gambia | Influenza | HBM | n/s |
| Chen 2015 (37) | Cross sectional | 1300 | 6 months-3years | Jul-Aug 2011 | Taiwan |  | HBM | n/s |
| Cheung 2015 (38) | Cross sectional | 2755 | 5-13 years | Sep 2009 &  Sep 2010 | United States | Influenza | HBM | Preferred language of English, Spanish, Chinese, or Vietnamese |
| Chau 2017 (39) | Cross sectional | 623 | N/S | May-Aug 2013 | China | influenza | HBM | Survey in Chinese |
| He 2015 (40) | Cross sectional | 335 | 6month-3 years | 2013 | China | Influenza | HBM | n/s |
| Hwang 2017 (41) | Cross sectional | 638 | 6-59 months | 2017 | South Korea | Influenza | HBM | n/s |
| Lau 2013 (44) | Cross sectional | 401 | 6-23 months | May-June 2006 | China | Influenza | HBM | n/s |
| Mergler 2013 (46) | Cross sectional | 1367 | School age | 2002-2003 & 2005 | United States | All childhood vaccines | HBM | n/s |
| O'Leary 2015 (48) | Cross sectional | 288 | 2-17 years | Apr-June 2012 | United States | Influenza | HBM | n/s |
| Scheuerman 2017 (56) | Cross sectional | 217 | 6 months-18 years | Aug-Oct 2011 & Sept-Oct 2012 | Israel | Influenza | HBM | Written in Hebrew |
| Tsuchiya 2016 (54) | Cross sectional | 226 | N/S | 2011 | Japan | Mumps | HBM | n/s |
| Tsuchiya 2014 (51) | Cross sectional | 220 | N/S | 2009-2010 | Japan | Influenza | HBM | n/s |
| Saitoh 2017 (50) | Cluster-randomized controlled-trial | 160 | <0 years | Sep 2014-Feb 2015 | Japan | All childhood vaccines | HBM and IBM | n/s |
| Wu 2014 (53) | Cross sectional | 118 | N/S | Jul-10 | China | H1N1 influenza | HBM and study conducted by Mok et al | n/s |
| Kempe 2014 (43) | Cross sectional | 699 | N/S | 2010 | United States | Influenza | HBM, survey instrument pilot tested | English and Spanish |
| Malosh 2014 (45) | Cross sectional | 778 | <18 years | 2010 | United States | Influenza | Adapted from research conducted during the 2009 H1N1 pandemic and HBM | n/s |
| Janks 2012 (42) | Cross sectional | 149 | 4-7 years | 2009 | United Kingdom | H1N1 influenza | Based on previous literature and HBM | n/s |
| Paek 2015 (49) | Cross sectional | 1017 | <12 years | 2014 | Korea | All childhood vaccines | Based on previous literature and HBM | n/s |
| Morin 2012 (47) | Cross sectional | 343 | N/S | Feb-11 | Canada | Rotavirus | Multidisciplinary team developed the questionnaire, checked with experts and piloted, HBM | n/s |
| Leonard 2017 (32) | Cross sectional | 243 | <6 years |  | United States | Measles, Mumps, Rubella | Parental Attitudes toward MMR Vaccine and Trust in Medical, Authority and HBM | no |
| Wagner 2017 (52) | Cross sectional | 619 | 8 months-7 years | May-Jun 2014 | China | Measles, Pneumonia, Meningitis | Previous literature and previous study by author and HBM | Survey in Chinese |
| Ben Natan 2016 (36) | Cross sectional | 200 | <12 years | Jan-Mar 2015 | Israel | Influenza | Scale designed by Chen et al. (2011) and HBM | Translated into Arabic |
| Schollin 2017 (55) | Cross sectional | 1063 | <12 weeks | Sep-Oct 2014 | Sweden | Rotavirus | Previous questionnaire used in a Canadian study (HBM) and experts | Translated in Swedish, English, Arabic, Polish, Spanish, Turkish and Somali |
| Peleg 2015 (57) | Cross sectional | 273 | <18 years | Sep-Oct 2011 | Israel | Influenza | Previous study of compliance to vaccinate children (based on HBM) | Written in Hebrew |
| Atkinson 2015 (35) | Cross sectional | 54 | <3 months | Nov 2013- Jul 2014 | Canada | All childhood vaccines | Piloted | n/s |
| Abu-rish 2016 (75) | Cross sectional | 317 | <5 years | Dec 2015 - Apr 2016 | Jordon | Influenza | Previous literature, experts and piloted | n/s |
| Chow 2012 (76) | Cross sectional | 431 | 6 months-5yrs | Nov-Dec 2009 | Australia | Influenza | Previous literature, qualitative interviews and piloted | n/s |
| Giambi 2018 (77) | Cross sectional | 3130 | 16-36 months | 2016 | Italy | All childhood vaccines | Previous literature and piloted | n/s |
| Smith 2017 (78) | Cross sectional | 1001 | 2-7 years | 2016-2017 flu season | United Kingdom | Influenza | Previous literature and piloted | n/s |
| Legesse 2015 (79) | Cross sectional | 591 | 12-23 months | Dec 2012- Jan 2013 | Ethiopia | All childhood vaccines | Previous literature | Translated into local language |
| Low 2017 (80) | Cross sectional | 332 | 6 month-5years | Dec 2015- Mar 2016 | Singapore | All childhood vaccines | Previous literature | n/s |
| Nowak 2017 (81) | Cross sectional | 1000 | <5 years | Nov-Dec 2014 | United States | All childhood vaccines | Previous literature | n/s |
| Reavis 2017 (82) | Pre-post intervention | 585 | <18 years | Jun-14 | United States | All childhood vaccines | Previous literature | n/s |
| Tam 2015 (83) | Cross sectional | 3484 | <12 years | Sep-Nov 2013 | Hong Kong | Varicella | Previous literature | n/s |
| Weiss 2016 (84) | Cross sectional | 189 | <36 months | 2011 | Switzerland | Measles | Previous literature and previous qualitative study | n/s |
| Young 2015 (85) | Cross sectional | 49 | N/S | N/S | United States | All childhood vaccines | Previous literature | n/s |
| Frawley 2018 (86) | Cross sectional | 429 | <6 years | N/S | Australia | All childhood vaccines | Previous literature, Larson et al. and piloted | n/s |
| Boes 2017 (87) | Cross sectional | 518 | N/S | May-June 2015 | Germany | Influenza | Previous literature and experts | n/s |
| Liao 2016 (88) | Longitudinal study | 1226 | 6 months - 6 years | Aug -Oct 2012 & March 2013 | China | All childhood vaccines | Previous literature and experts |  |
| Livni 2017 (89) | Cross sectional | 186 | <18 years | Sep - Oct 2012 | Israel | Influenza | Previous literature | Questionnaire written in Hebrew |
| MacDonald 2014 (90) | Case control study | 461 | 2 years | May 2008 - April 2009 | Canada | All childhood vaccines | Previous literature (only used previously validated studies) and experts | n/s |
| Rao 2018 (91) | Cross sectional | 1001 | >6 months | Oct 2014-Mar 2015 | United States | Influenza | Previous literature, experts and piloted | n/s |
| Brown 2014 (92) | Cross sectional | 2065 | <14yrs | Jun-10 | United States | Influenza | Previous literature and focus groups and pretest | n/s |
| Veldwijk 2014 (93) | Cross sectional | 466 | 6 weeks | N/S | Netherlands | Rotavirus | Previous literature, experts, parents and piloted | n/s |
| My 2017 (94) | Cross sectional | 452 | <18 years | 2012 | Australia | All childhood vaccines | Previous standardised national and international surveys | n/s |
| Luthy 2013 (27) | Cross sectional | 801 |  |  | United States | All childhood vaccines | Questionnaire adapted from Utah State Department of Health (USDOH) exemption questionnaire, expert review |  |
| Shrestha 2016 (29) | Case control study | 262 | 12-23 months | Sept-Nov 2014 | Nepal | All childhood vaccines | Adapted from Nepal Demographic Health Survey 2011, Immunization Coverage Cluster Survey, previous literature and experts | Translated in to Nepali |
| Schonburger 2012 (28) | Cross sectional | 3041 | <4 years | 2002-2005 | Germany | All childhood vaccines | National immunisation survey | Interviews in German |
| LaVail 2013 (26) | Cross sectional | 376 | <6 years | Sept-Oct 2010 | United States | All childhood vaccines | 2010 HealthStyles; annual validated mail-panel survey of adults living in the US | n/s |
| Gaudino 2012 (95) | Retrospective cohort | 2900 | School aged | 2004-2005 | United States | All childhood vaccines | Based on previous study by authors, piloted | Spanish and other language translations |
| Smith 2015 (30) | Cross sectional | 12259 | 19-35 months | 2010-2013 | United States | Measles | National Immunisation Survey and HBM | n/s |
| Garcia 2014 (96) | Cross sectional | 4802 | <5 years | May-June 2010 | Colombia | All childhood vaccines | Conducted a pre-study evaluation of target population, focus groups | n/s |
| Wang 2016 (97) | Cross sectional | 1996 | 12-17 years | Feb-Nov 2014 | China | All childhood vaccines | Earlier qualitative studies on Hong Kong and mainland Chinese immigrant parents and previous literature | n/s |
| Walsh 2015 (31) | Case control study | 308 | 2 years | Jul-Sep 2001 | United Kingdom | Measles, Mumps, Rubella | Evidence-based information pack on MMR, by Welsh Assembly Government | n/s |
| Moulsdale 2017 (98) | Cross sectional | 86 | 3-11 years | 2014 | United Kingdom | Influenza | Informed by an earlier postal, cross-sectional survey in Bristol | n/s |
| Weiner 2015 (64) | Cross sectional | 200 | <0 years | Jun-Sep 2014 | United States | All childhood vaccines | The survey instrument was developed using or adapting existing questions whenever possible | n/s |
| Thorpe 2012 (63) | Cross sectional | 124 | 0-18 years | May-10 | United States | All childhood vaccines | Triandis Model of the Theory of Reasoned Action | n/s |
| Umeh 2018 (33) | Cross sectional | 396 | N/S | N/S | Nigeria | Polio | VACSATC (Vaccine Safety, Attitudes, Training and Communication) & SUBI (Subjective Wellbeing Inventory) | Translated to Hausa |
| Kettunen 2017 (99) | Cross sectional | 84 | N/S | 2015 | United States | N/S | PEN-3-Cultural Model, based on previous study developed by experts and piloted | Designed with input from Amish community |
| Hilyard 2014 (100) | Cross sectional | 684 | <18 years | 2010 | United States | H1N1 influenza | Developed by experts | n/s |
| Masadeh 2014 (101) | Cross sectional | 506 |  | Jun-Aug 2012 | Jordon | All childhood vaccines | Developed by research team, reviewed by colleagues, pilot tested | n/s |
| Larson 2015 (102) | Cross sectional | 5354 | <5 years | 2014 | United States, India, Nigeria, Pakistan, United Kingdom | All childhood vaccines | Developed by Vaccine Confidence Project | Conducted in the relevant local language |
| Garg 2018 (103) | Cross sectional | 160 | <3 years | N/S | United States | All childhood vaccines | Cognitive interviews, self-developed scales | n/s |
| Rogers 2014 (104) | Cross sectional | 51 | Pre-school aged | N/S | United States | All childhood vaccines | Based on principles of good survey and question design | n/s |
| Angadi 2013 (105) | Cross sectional | 155 | 12-23 months | Oct-Nov 2011 | India | All childhood vaccines | n/s | n/s |
| Bakhache 2013 (106) | Cross sectional | 2460 | 0-23 months | Sep- Nov 2011 | Australia, Canada, France, Germany, Spain, Sweden, United Kingdom | All childhood vaccines | n/s | Translated into local languages |
| Bamatraf 2018 (107) | Cross sectional | 400 | N/S | Dec 2014 - Mar 2015 | Yemen | All childhood vaccines | n/s | Some language changed into local words |
| Bazzano 2012 (108) | Cross sectional | 197 | <18 years | Mar- Sep 2007 | United States | All childhood vaccines | n/s | Translated into Spanish |
| Blyth 2014 (109) | Cross sectional | 2576 | N/S | Influenza seasons 2008–2012 | Australia | Influenza | n/s | n/s |
| Braczkowska 2018 (110) | Cross sectional | 1239 | 6-13 years | 2016 | Poland | All childhood vaccines | n/s | n/s |
| Bukhsh 2018 (111) | Cross sectional | 532 | >6 months | N/S | Pakistan | Influenza | n/s | n/s |
| Buyuktiryaki 2014 (112) | Cross sectional | 625 | 6-18 years | Apr-July 2010 | Turkey | A/H1N1 influenza | n/s | n/s |
| Cacciatore 2016 (113) | Pre-post questionnaire | 1855 | <5 years | Nov-Dec 2014 & May-June 2015 | United States | All childhood vaccines | n/s | n/s |
| Campbell 2017 (114) | Cross sectional | 1792 | 2 months-5 years | Jan-Apr 2015 | United Kingdom | All childhood vaccines | n/s | n/s |
| Dubé 2014 (65) | Cross sectional | 236 | 6 months & 17 years | Oct-Dec 2012 | Canada | Influenza | n/s | n/s |
| Esposito 2018 (115) | Cross sectional | 114 | N/S | Nov 2014-April 2015 | Italy | All childhood vaccines | n/s | n/s |
| Gunduz 2014 (116) | Cross sectional | 285 | 1-16 years | 2012 | Turkey | Influenza | n/s | n/s |
| Healy 2014 (117) | Cross sectional | 401 | <18 years | N/S | United States | All childhood vaccines | n/s | English and Spanish |
| How 2016 (118) | Cross sectional | 200 | <5 years | 2014 | Singapore | Pneumococcal | n/s | Translated into major languages of Singapore |
| Idoko 2016 (119) | Pre-post survey | 587 | ~4 months | 2015 | The Gambia | Polio | n/s | n/s |
| Kalucka 2016 (120) | Cross sectional | 78 | <7 years | N/S | Poland | All childhood vaccines | n/s | n/s |
| Kaya 2017 (121) | Cross sectional | 81 | N/S | N/S | Turkey | Influenza | n/s | n/s |
| Kelley 2015 (122) | Cross sectional | 379 | <5 years | 2010 | United States | All childhood vaccines | n/s | n/s |
| Kim 2013 (123) | Cross sectional | 500 | N/S | 2012 | South Korea | Tetanus, diphtheria, and pertussis | n/s | n/s |
| Lee 2016 (124) | Case control study | 1253 | School age | 2002-2003 | United States | All childhood vaccines | n/s | n/s |
| Lehmann 2017 (125) | Cross sectional | 1615 | 3 months - 3.5 years | September 2015 | Netherlands | All childhood vaccines | n/s | n/s |
| McCauley 2012 (126) | Cross sectional | 1500 | 6-23 months | March-April 2010 | United States | All childhood vaccines | n/s | n/s |
| Michael 2014 (127) | Cross sectional | 148 | N/S | Oct 2012 | Nigeria | Polio | n/s | Interviews conducted in local language |
| Mollema 2012 (128) | Cross sectional (x2) | 7085 & 2934 | N/S | 1995-1996 &  2006-2007 | Netherlands | All childhood vaccines | n/s | n/s |
| Mrozek-Budzyn 2016 (129) | Cross sectional | 154 | N/S | 2014-2015 | Poland | All childhood vaccines | n/s | n/s |
| Muhsen 2012 (130) | Case control study | 430 | 2-25years & 2-5years | 2007 | Israel | All childhood vaccines | n/s | yes |
| Oria 2013 (66) | Before and After study | 5284 & 5755 | 6 months-10 years | Jun &Sep 2010 | Kenya | Influenza | n/s | n/s |
| Oskarsson 2015 (131) | Cross sectional | 5584 | N/S | 2013-2014 | Iceland | All childhood vaccines | n/s | n/s |
| Pelullo 2014 (132) | Cross sectional | 1039 | N/S | Jan-April 2013 | Italy | All childhood vaccines | n/s | n/s |
| Perinet 2018 (133) | Cross sectional | 3604 | 2 years | Mar-13 | Canada | Measles | n/s | n/s |
| Preza 2017 (134) | Cross sectional | 288 | 2-4 months | Dec 2014-July 2015 | Albania | All childhood vaccines | n/s | n/s |
| Rabinowitz 2016 (135) | Cross sectional | 367 | N/S | N/S | United States | All childhood vaccines | n/s | n/s |
| Ramprasad 2017 (136) | Cross sectional | 456 | 6 months-15 years | Sep-Oct 2012 | India | Influenza | n/s | n/s |
| Sohail 2015 (137) | Cross sectional | 200 | <16 months | n/s | Pakistan | All childhood vaccines | n/s |  |
| Thors 2014 (138) | Cross sectional | 253 | 2-11 years | 2012-2013 | United Kingdom | Influenza | n/s | n/s |
| Van Lier 2016 (139) | Cross sectional | 491 | 0-4 years | 2012 | Netherlands | Varicella | n/s | n/s |
| Vezzosi 2017 (140) | Cross sectional | 414 | N/S | May-Jun 2015 | Italy | Varicella | n/s | n/s |
| Weston 2017 (141) | Cross sectional | 85 | 0-15 years | 2010 | United Kingdom | H1N1 influenza | n/s | n/s |
| Wolff 2014 (142) | Cross sectional | 99 | N/S | Aug 2012-Feb 2013 | United States | All childhood vaccines | n/s | Interpreters used for non-English-speaking participants |
